# Gender-related differences in clinicopathological characteristics and renal outcomes of Chinese patients with IgA nephropathy

**DOI:** 10.1186/s12882-018-0829-1

**Published:** 2018-02-07

**Authors:** Wei Deng, Xiaojun Tan, Qian Zhou, Zhen Ai, Wenting Liu, Wei Chen, Xueqing Yu, Qiongqiong Yang

**Affiliations:** 1 Department of Nephrology, The First Affiliated Hospital, Sun Yat-sen University, Key Laboratory of Nephrology, Ministry of Health and Guangdong Province, Guangzhou, Guangdong 510080 China; 2 Department of Nephrology, Kaiping Central Hospital, Jiangmen, 529300 China; 3 Clinical Research Centre, The First Affiliated Hospital, Sun Yat-sen University, Guangzhou, 510080 China

**Keywords:** Gender, Renal survival, Risk factor, IgA nephropathy

## Abstract

**Background:**

The prognostic effect of gender on immunoglobulin A nephropathy (IgAN) is not clear. We explored gender-related differences in clinicopathological features and renal outcomes in IgAN.

**Methods:**

This was a single-centre retrospective study. Patients were divided into two groups according to gender. The clinicopathological features at biopsy and renal outcomes during the follow-up were collected and analysed. Renal outcomes were defined as the doubling of baseline serum creatinine and end-stage renal disease (ESRD) (estimated glomerular filtration rate < 15 mL/min/1.73 m^2^, dialysis, or renal transplantation). The prognostic effects of gender were evaluated by Cox regression models.

**Results:**

A total of 988 eligible IgAN patients were enrolled, and the ratio of males to females was 1:1.4. Compared with female patients, male patients had worse renal function, greater proteinuria, a higher prevalence of hypertension, hypertriglyceridaemia and hyperuricaemia, and more severe segmental sclerosis and tubular atrophy/interstitial fibrosis. However, haematuria occurred more frequently in female patients. During a median follow-up time of 48.6 (34.7, 62.7) months, no differences in renal survival rates were noted between the male and female groups. Multivariable Cox regression analyses revealed that gender was not a significant risk factor for renal outcomes after frequency matching of baseline eGFR and serum uric acid (SUA) levels. In addition, male and female patients shared similar risk factors, including a low eGFR and increased proteinuria and segmental sclerosis. In males, however, an elevated proportion of global glomerulosclerosis was also a poor prognostic factor for renal survival.

**Conclusions:**

Male IgAN patients presented with worse clinicopathologic features than female patients, but no significant differences were observed in long-term renal survival between male and female patients by eGFR- and SUA level-matching.

## Background

Immunoglobulin A nephropathy (IgAN) is a common glomerulonephritic disease that is one of the major causes of end-stage renal disease (ESRD) [1]. The prevalence of IgAN in male and female patients differs geographically. The ratios of male to female patients range from less than 2:1 in Asians to as high as 6:1 in Europe and the United States [2–4]. Poor prognostic factors for renal progression in IgAN have been identified, including hypertension, heavy proteinuria, and severe histopathological abnormalities [5–7]. However, the differences in renal progression between male and female IgAN patients are controversial. Several studies have found that renal progression occurs faster in males than in females [5, 8], whereas other studies have found either no gender differences [9, 10] or a greater risk of renal progression in females [11]. The impact of gender on the progression of IgAN remains uncertain. Meanwhile, few studies have specifically described the associations between gender and renal outcomes [8, 12]. To better understand these associations in IgAN patients, we conducted a retrospective study of a large number of Chinese patients with biopsy-diagnosed primary IgAN.

## Methods

### Study design and participants

This was a single-centre retrospective study. All patients with biopsy-diagnosed primary IgAN who were recorded in the IgAN Database of The First Affiliated Hospital, Sun Yat-sen University from 1 January 2006 to 31 December 2011 were considered. The inclusion criteria were as follows: biopsy-diagnosed primary IgAN, age ≥ 14 years, adequate biopsy sample containing ≥10 glomeruli, and willingness to sign the informed consent form. Patients were excluded if they presented with secondary causes of mesangial IgA deposition (such as hepatitis B-related nephritis, Henoch-Schonlein purpura nephritis, lupus nephritis, transplantation-related IgAN, etc), ESRD [estimated glomerular filtration rate (eGFR) < 15 mL/min/1.73 m^2^], or a follow-up duration < 12 months. Patients who suffered from kidney stone, urological malignancies, or menstruating females were also excluded.

### Data collection

Demographic and clinicopathologic data were collected at biopsy. Clinical parameters included gender, age, blood pressure (BP), hypertension (defined as systolic BP ≥140 mmHg and/or diastolic BP ≥90 mmHg or requirement for anti-hypertensive therapy), haemoglobin, total protein, serum albumin, total cholesterol, triglycerides, hypercholesterolaemia (defined as serum total cholesterol ≥5.2 mmol/L), hypertriglyceridaemia (defined as serum triglycerides ≥1.7 mmol/L), serum uric acid (SUA), hyperuricaemia (defined as SUA > 420 μmol/L in males, and > 360 μmol/L in females), serum creatinine (Scr), blood urine nitrogen (BUN), eGFR (estimated using the Modification of Diet in Renal Disease (MDRD) equation) [13], proteinuria (defined as 24-h urine protein excretion), gross haematuria (glomerular was distinguished from non-glomerular haematuria by examining urine sediment with a phase contrast microscope), microscopic haematuria (defined as > 3 red blood cells in a high-power field on microscopic evaluation), and medication regimen.

Renal biopsy specimens were examined by light, immunofluorescence and electron microscopy (EM) and graded according to the Oxford classification system, which includes mesangial hypercellularity (M), endocapillary hypercellularity (E), segmental sclerosis (S), and tubular atrophy/interstitial fibrosis (T) [14]. The pathological features also included global glomerulosclerosis, crescent formation, capillary loop necrosis, interstitial inflammation and vascular lesions. All renal biopsy results were reviewed independently by two renal pathologists.

### Outcomes of interest

The primary endpoint was renal outcome comprising the doubling of baseline Scr and ESRD (eGFR< 15 mL/min/1.73 m^2^, dialysis, or renal transplantation). The renal outcomes were collected from the time of biopsy to the end of the follow-up period.

### Statistical analyses

Continuous data with a normal distribution were expressed as the mean ± SD and compared by independent sample t tests. Continuous data with a skewed distribution were expressed as the median (inter-quartile range, IQR) and were analysed by Wilcoxon rank sum tests. Categorical variables were summarized as frequencies and percentages, and comparisons were conducted using chi-squared tests. The cumulative renal survival rates were estimated by the Kaplan-Meier method, and the log-rank test was applied for comparisons. The variables affecting renal outcomes were identified by univariable and multivariable Cox proportional hazards models. The proportional hazard assumption was evaluated in each multivariable Cox model, and if violated, time-dependent covariates were used for adjustments. The results are expressed as a hazard ratio (HR) and 95% confidence interval (CI). Sensibility analyses were conducted using frequency matching for eGFR (< 60.0, 60.0–89.9, and ≥90.0 mL/min/1.73 m^2^) and SUA levels (< 316, 316–419, 420–524, 525–629, and ≥630 μmol/L for males; < 271, 271–359, 360–449, 450–539, and ≥540 μmol/L for females) to balance significant differences between gender groups and then to evaluate the association of gender with renal survival. Due to inherent physical differences in SUA levels between genders, use of the same values to group both male and female patients could possibly exaggerate the clinical significance of SUA in the different genders. Therefore, we divided the patients into different SUA groups based on the normal SUA levels of the two genders. The group intervals were equal to one-fourth of the normal SUA levels in the two genders: 105 μmol/L in males and 90 μmol/L in females. All *P*-values were two-tailed, and a value < 0.05 was considered statistically significant. Data analysis was performed using SPSS (version 13; SPSS Inc., Chicago, IL).

## Results

### Clinicopathological features of IgAN patients

A total of 988 IgAN patients were analysed in this study. The ratio of males to females was 1:1.4, with a median age of 32 (26, 39) years at biopsy. The results of comparisons of clinical and histological parameters between male and female patients are summarized in Table 1. Compared with female patients, male IgAN patients exhibited more severe clinical manifestations, including higher levels of serum creatinine, BUN and SUA; a lower eGFR; heavier proteinuria; and higher proportions of hypertension, hypertriglyceridaemia and hyperuricaemia (*P* < 0.05). In female patients, the most common renal manifestation was haematuria, including a higher frequency of gross haematuria and more severe microscopic haematuria (*P* < 0.01). Additionally, male patients had worse histological lesions, including more S, T and interstitial inflammation (*P* < 0.05). There were no differences in the proportion of global glomerulosclerosis, crescent formation, or the degree of M and E lesions between male and female patients (*P >* 0.05). No significant differences were observed in the use of ACEi/ARB, corticosteroids or immunosuppressive agents between the genders. The frequency of allopurinol usage was higher in males than in females (10.7% vs. 1.8%).

**Table 1:** Clinicopathologic characteristics of IgAN patients of different genders

Variable	Total (*n* = 988)	Males (*n* = 417)	Females (*n* = 571)	*P-*value
Age (years)	32 (26, 39)	31 (24, 38)	32 (27, 39)	0.003
Systolic blood pressure (mmHg)	123 (112, 136)	128 (119, 140)	119 (110, 133)	< 0.001
Diastolic blood pressure (mmHg)	79 (70, 88)	80 (71, 89)	78 (70, 87)	< 0.001
Hypertension (n [%])	331 (33.5)	168 (40.3)	163 (28.5)	< 0.001
Proteinuria (g/d)	0.58 (0.30, 1.21)	0.69 (0.33, 1.47)	0.53 (0.27, 1.05)	< 0.001
Microscopic haematuria (n [%])				0.006
<++	590 (60.0)	271 (65.1)	319 (56.3)	
≥++	393 (40.0)	145 (34.9)	248 (43.7)	
Gross haematuria (n [%])	280 (28.3)	82 (19.7)	198 (34.7)	< 0.001
Haemoglobin (g/L)	127.0 (115.0, 140.0)	139.0 (128.0, 151.0)	121.0 (111.0, 129.0)	< 0.001
Total protein (g/L)	67.0 (61.0, 71.0)	67.0 (62.0, 72.0)	66.0 (61.0, 71.0)	0.613
Albumin (g/L)	40.0 (36.5, 42.9)	41.0 (37.4, 43.0)	39.0 (36.0, 42.0)	< 0.001
Cholesterol (mmol/L)	4.97 (4.2, 5.9)	4.9 (4.2, 5.9)	5.0 (4.3, 5.9)	0.932
Hypercholesterolaemia (n [%])	397 (42.0)	168 (41.7)	229 (42.3)	0.894
Triglycerides (mmol/L)	1.25 (0.87, 1.90)	1.39 (0.98, 2.00)	1.15 (0.77, 1.81)	< 0.001
Hypertriglyceridaemia (n [%])	282 (29.8)	136 (33.7)	146 (26.9)	0.026
SUA (μmol/L)	348 (269, 429)	405 (343, 492)	295 (239, 373)	< 0.001
Hyperuricaemia (n [%])	322 (34.7)	174 (43.9)	148 (27.9)	< 0.001
Scr (mmol/L)	80.0 (61.0, 116.5)	102.0 (81.0, 161)	64.0 (55.0, 89.0)	< 0.001
BUN (mmol/L)	5.4 (4.4, 7.2)	6.2 (4.8, 8.1)	4.9 (4.1, 6.3)	< 0.001
eGFR (mL/min/1.73 m^2^)	95.5 (59.9, 123.2)	80.8 (42.9.1, 107.4)	111.3 (71.9, 133.7)	< 0.001
Mean proportion of global glomerulosclerosis (%)	11.1 (0.0, 30.8)	13.3 (0.0, 35.7)	10.0 (0.0, 27.8)	0.205
Crescent (n [%])	434 (43.9)	195 (46.8)	239 (41.9)	0.136
Mesangial hypercellularity (M1) (n [%])	524 (54.0)	225 (55.3)	299 (53.1)	0.514
Endocapillary hypercellularity (E1) (n [%])	193 (20.4)	77 (19.4)	116 (21.1)	0.568
Segmental sclerosis (S1) (n [%])	447 (46.3)	204 (50.4)	243 (43.3)	0.031
Tubular atrophy/interstitial fibrosis (T) (n [%])				0.001
T0	711 (73.5)	273 (67.4)	438 (77.9)	
T1	227 (23.5)	119 (29.4)	108 (19.2)	
T2	29 (3.0)	13 (3.2)	16 (2.8)	
Interstitial inflammation (n [%])				0.020
None~mild	818 (82.8)	329 (78.9)	489 (85.6)	
Moderate	151 (15.3)	79 (18.9)	72 (12.6)	
Severe	19 (1.9)	9 (2.2)	10 (1.8)	
ACEi/ARB (n [%])	781 (79.4)	335 (80.9)	446 (78.2)	0.338
Corticosteroids (n [%])	293 (29.8)	137 (33.1)	156 (27.4)	0.057
Immunosuppressive agents (n [%])	22 (2.3)	11 (2.7)	11 (2.0)	0.515
Allopurinol (n [%])	54 (5.5)	44 (10.7)	10 (1.8)	<0.001

Continuous variables: mean ± SD; Continuous skewed variables: median (interquartile range); Categorical variables: frequency (percentage)

*SUA* serum uric acid, *Scr* serum creatinine, *eGFR* estimated glomerular filtration rate, *BUN* blood urea nitrogen, *ACEI* angiotensin converting enzyme inhibitor, *ARB* angiotensin II receptor blocker

ACEi/ARB indicates exposure to ACEI, ARB, or both. Immunosuppressive agents included MMF, CTX, CsA, and FK506

### Renal survival of IgAN patients according to gender

**Fig. 1:**
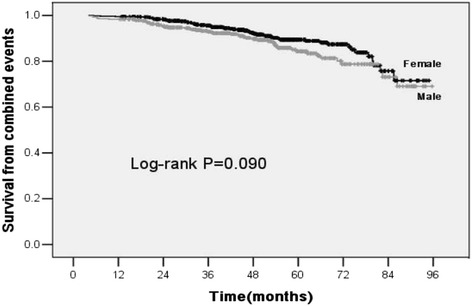
Renal survival curves of male and female IgAN patients. No significant difference was observed in the renal survival rate of combined events (doubling of baseline Scr and ESRD) between the different genders of IgAN patients

After a median follow-up period of 48.6 (34.7, 62.7) months, 107 (10.8%) patients (48.6% male vs 51.4% female) reached renal outcomes. A total of 16 (1.6%) patients achieved doubling of Scr, and 91 (9.2%) patients developed ESRD. Kaplan-Meier curves showed that the renal survival rates calculated from the combined events at 3, 5, and 7 years were 92.9%, 84.3%, and 73.1% in males and 95.6%, 89.4%, and 75.8% in females, respectively. No significant difference was observed in cumulative renal survival rates between male and female IgAN patients (log-rank test *P* = 0.090) (Fig. 1).

Univariate analysis showed no significant differences in renal outcomes between males and females (HR: 1.39, *P* = 0.091). Next, we constructed multivariable Cox models of gender adjusted for clinical factors and pathologic features, but no significant differences in renal outcomes between male and female IgAN patients were observed after adjustments for age and hypertension in Model 1 (HR: 1.12, *P* = 0.584), after adding pathologic features (proportions of global glomerulosclerosis, degrees of S and T lesions) in Model 2 (HR: 0.95, *P* = 0.824), or after adding pathologic features and clinical parameters (albumin and proteinuria) in Model 3 (HR: 0.97, *P* = 0.876). However, male gender was shown to be a protective factor when fully adjusting for eGFR and the SUA level in Model 4 (HR: 0.59, *P* = 0.029) (Table 2).

**Table 2:** The association of gender with renal survival by Cox regression analysis before and after frequency matching of SUA levels and eGFR

Model	Hazard ratios (95% CI)	*P-*value
Before matching		
Univariate	1.39 (0.95, 2.03)	0.091
Model 1: age, hypertension	1.12 (0.76, 1.65)	0.584
Model 2: Model 1+ proportion of global glomerulosclerosis, S1, T1, T2	0.95 (0.62, 1.46)	0.824
Model 3: Model 2 + Albumin, Proteinuria	0.97 (0.62, 1.50)	0.876
Model 4: Model 3 + eGFR, SUA	0.59 (0.36, 0.95)	0.029
After matching		
Univariate	0.75 (0.48,1.18)	0.220
Model 1: age, hypertension	0.62 (0.39,1.00)	0.052
Model 2: Model 1 + proportion of global glomerulosclerosis, S1, T1, T2	0.73 (0.43,1.24)	0.248
Model 3: Model 2+ Proteinuria	0.72 (0.42,1.23)	0.230

*CI* confidence interval, *eGFR* estimated glomerular filtration rate, *SUA* serum uric acid, *S* segmental sclerosis, *T* tubular atrophy/interstitial fibrosis

Significant differences were found in the SUA level and eGFR between female and male patients (*P* < 0.001). To eliminate biases due to significant intergroup differences, we conducted sensibility analyses by frequency matching the eGFR (< 60.0, 60.0–89.9, and ≥90.0 mL/min/1.73 m^2^) and SUA levels (< 316, 316–419, 420–524, 525–629, and ≥630 μmol/L for males; < 271, 271–359, 360–449, 450–539, and ≥540 μmol/L for females) between male and female groups and then evaluating the association between gender and renal survival. As shown in Table 3, after controlling for these confounding factors (baseline eGFR and SUA) by frequency matching, no significant difference was observed in long-term renal survival between male and female IgAN patients (HR: 0.72, *P* = 0.230) (Table 2).

**Table 3:** Distribution of eGFR and SUA levels before and after frequency matching in the current study

Variables			Males		Females	*P-*value
			n = 417		n = 571	
Before matching (n = 988)	eGFR (mL/min/1.73 m^2^)		80.8 (42.9, 107.4)		111.3 (71.9, 133.7)	< 0.001
	eGFR group (n[%])	< 60.0	139 (33.3)	< 60.0	95 (16.6)	< 0.001
		60.0–89.9	112 (26.9)	60.0–89.9	98 (17.2)	
		≥90.0	166 (39.8)	≥90.0	378 (66.2)	
	SUA (μmol/L)		405 (343, 492)		295 (239, 373)	< 0.001
	SUA group (n[%])	< 316	77 (19.4)	< 271	214 (40.3)	< 0.001
		316–419	145 (36.6)	271–359	166 (31.3)	
		420–524	100 (25.3)	360–449	99 (18.6)	
		525–629	56 (14.1)	450–539	36 (6.8)	
		≥630	18 (4.5)	≥540	16 (3.0)	
	Hyperuricaemia (n [%])		174 (43.9)		148 (27.9)	< 0.001
After matching (*n* = 678)			*n* = 329		*n* = 349	
	eGFR (mL/min/1.73 m^2^)		88.8 (59.0, 115.3)		91.1 (61.0, 118.2)	0.353
	eGFR group (n [%])	< 60.0	84 (25.5)	<60.0	83 (23.8)	0.812
		60.0–89.9	83 (25.2)	60.0–89.9	86 (24.6)	
		≥90.0	162 (49.2)	≥90.0	180 (51.6)	
	SUA (μmol/L)		400.0 (332.0, 469.0)		328.0 (289.0, 404.0)	< 0.001
	SUA group (n [%])	< 316	67 (21.3)	< 271	59 (17.8)	0.601
		316–419	118 (37.5)	271–359	143 (43.2)	
		420–524	88 (27.9)	360–449	86 (26.0)	
		525–629	33 (10.5)	450–539	32 (9.7)	
		≥630	9 (2.9)	≥540	11 (3.2)	
	Hyperuricaemia (n [%])		130 (41.3)		126 (38.1)	0.422

*eGFR* estimated glomerular filtration rate, *SUA* serum uric acid

SUA group intervals were equal to one-fourth of the normal SUA levels of the different genders

In addition, to identify the associated risk factors of composite renal outcomes (doubling of baseline Scr and ESRD) in males and females with IgAN, univariate and multivariate analyses were performed. As shown in Tables 4 and 5, Cox regression analysis revealed similar risk factors in male and female IgAN patients, including low eGFR, high levels of proteinuria and increased S1 lesions (*P* < 0.05). However, an increased proportion of global glomerulosclerosis was significant only in male patients (HR: 1.02, *P* = 0.028).

**Table 4:** Associated risk factors of renal survival in male patients

	Univariate		Multivariate	
Variables	HR (95% CI)	*P-*value	HR (95% CI)	*P-*value
Age (per 1-year increase)	1.00 (0.98, 1.03)	0.880	0.97 (0.94, 1.01)	0.139
Hypertension (vs. no)	2.43 (1.40, 4.23)	0.002	0.62 (0.32, 1.19)	0.150
Proteinuria (1 g/d increase)	1.28 (1.14, 1.43)	< 0.001	1.22 (1.02, 1.46)	0.032
eGFR (1 mL/min/1.73 m^2^ increase)	0.95 (0.94, 0.96)	< 0.001	0.95 (0.94, 0.97)	< 0.001
Proportion of global glomerulosclerosis (1% increase)	1.05 (1.04, 1.06)	< 0.001	1.02 (1.00, 1.04)	0.028
S1 (vs. S0)	3.60 (1.95, 6.61)	< 0.001	3.11 (1.49, 6.47)	0.002
T1 (vs. T0)	8.47 (4.25, 16.90)	< 0.001	0.83 (0.30, 2.28)	0.718
T2 (vs. T0)	41.65 (15.38, 112.79)	< 0.001	1.89 (0.50, 7.24)	0.351

*HR* hazard ratio, *CI* confidence interval, *eGFR* estimated glomerular filtration rate, *S* segmental sclerosis, *T* tubular atrophy/interstitial fibrosis

**Table 5:** Associated risk factors of renal survival in female patients

	Univariate		Multivariate	
Variable	HR (95% CI)	*P-*value	HR (95% CI)	*P-*value
Age (per 1-year increase)	0.99 (0.97, 1.02)	0.631	1.13 (0.92, 1.40)	0.242
Hypertension (vs. no)	4.85 (2.81, 8.36)	< 0.001	1.76 (0.85, 3.62)	0.126
Proteinuria (1 g/d increase)	1.76 (1.52, 2.04)	< 0.001	1.26 (1.03, 1.54)	0.023
eGFR (1 mL/min/1.73 m^2^ increase)	0.96 (0.95, 0.97)	< 0.001	0.98 (0.96, 0.99)	< 0.001
Proportion of global glomerulosclerosis (1% increase)	1.05 (1.04, 1.06)	< 0.001	1.10 (1.00, 1.21)	0.058
S1 (vs. S0)	4.93 (2.69, 9.02)	< 0.001	2.36 (1.42, 5.45)	0.012
T1 (vs. T0)	10.81 (5.75, 20.33)	< 0.001	1.75 (0.80, 3.81)	0.162
T2 (vs. T0)	31.43 (13.53, 73.02)	< 0.001	1.76 (0.58, 5.30)	0.318

*HR* hazard ratio, *CI* confidence interval, *eGFR* estimated glomerular filtration rate, *S* segmental sclerosis, *T* tubular atrophy/interstitial fibrosis

## Discussion

To date, only a few studies have evaluated differences in clinicopathologic features and the prognosis of IgAN between the different genders. In this study, we found that male IgAN patients presented with worse clinicopathological features than female patients. However, no significant difference was observed in long-term renal survival between male and female patients after frequency matching of baseline eGFR and SUA levels. In addition, we found that male and female patients shared similar risk factors, including low eGFR, heavy proteinuria and extensive segmental sclerosis, but the proportion of global glomerulosclerosis was a significant risk factor only in male patients.

According to our study, approximately 58.3% of the patients diagnosed with IgAN were females. The gender distribution of IgAN in China is similar to that in other nations in East Asia, with a male to female ratio of less than 2:1 [9, 15, 16], but it is different from that in Northern Europe and the United States, with a ratio as high as 6:1 [11, 17]. Variations in the gender distribution of IgAN may reflect differences in biopsy indications in different regions and could also be due to the diversity of genetic backgrounds.

In the clinicopathologic baseline data, we noticed the relatively worse condition of the male patients compared with the female patients, which is similar to previous reports. A study by Cattran DC et al. reported a higher mean arterial pressure (MAP) and lower corrected creatinine clearance (CrCl) in males than in females with IgAN [10]. In another study, Eiro et al. indicated that male gender was positively correlated with hypertension [18]. However, Tanaka K et al. more frequently observed a high level of urinary red blood cells (≥20/HPF) in female patients [19]. Our study not only confirmed these results but also found that male IgAN patients exhibited greater urinary protein excretion. In addition, we found that severe renal histological injury occurred even in male patients, including increased S, T and interstitial inflammation, which may be predictors of renal progression in IgAN. These findings suggest that male patients with IgAN disease, which was mainly mediated through worse clinicopathological characteristics, may have poorer renal outcomes.

However, the impact of gender on the progression of IgAN remains under debate. In a study by Goto M, male gender, rather than female gender, was associated with the risk of ESRD [5]. In addition, according to correlation analysis of the main prognostic risk factors affecting the progression of IgAN, Riispere Ž et al. concluded that IgA nephropathy progressed more rapidly in males compared with females [8]. The possible mechanisms underlying the renal protective role of female gender seem to be related to oestrogen [12]. However, other investigators have found either no gender-related differences or have observed women to be at a greater risk of a progressive loss of renal function. Donadio JV et al. reported contrary results for IgAN in female patients, pointing to worse disease outcomes [11]. Furthermore, a study performed by Cattran DC et al. showed no significant difference in long-term renal survival in IgAN between the different genders [10].

To our surprise, in our study, male gender predicted poorer outcomes in multivariable analysis, but male gender lost its prognostic value after frequency matching of baseline eGFR and SUA levels. The reason for this finding may be the possible confounding effects of baseline factors. Significant differences were observed in baseline eGFR and SUA levels between female and male patients. As shown in Table 1, a low eGFR and a high SUA level were more frequently observed in males than in females (*P* < 0.001). The major confounding factors in our study were imbalances in baseline eGFR and SUA levels. To control for the confounding effect of renal function on the association between gender and progression, eGFR- and Scr-matched and frequency matching analyses were performed. After frequency matching of clinically relevant factors, including eGFR and SUA levels, which are associated with renal outcomes, the gender difference lost its significance in multivariate analysis. Therefore, the potential risk effect of gender on renal outcomes may be due to these gender differences in the association between the eGFR and SUA levels and the progression of IgAN. The MDRD study showed slower renal disease progression in glomerular filtration rate (GFR) in women, as well as a significantly slower rate of GFR decline in women than in men [20, 21]. In general, women have lower average rates of creatinine generation, due to lower muscle mass and meat intake. Similarly, the SUA concentration increases with age and is physiologically higher in males than age-matched females [22]. The study by Nagasawa Y et al. indicated a gender difference with respect to the effects of uric acid on the progression of uric acid-induced kidney disease [23]. The mechanism of this gender difference in the association between the SUA level and the progression of IgAN remains unclear. Oestrogen suppresses the protein levels of the urate reabsorptive transporter (urate transporter1; URAT1) in the kidney, which results in an increase in uric acid excretion and a decrease in the uric acid level in the serum [24]. Moreover, Kolz et al. have found that the minor allele for rs734553 in SLC2A9 has a greater influence on reducing SUA levels in females and that the minor allele of rs2231142 in ABCG2 increases SUA levels more strongly in males compared with females [25].

Proteinuria, hypertension and renal function are important risk factors for IgAN [5, 7, 9, 16]. In addition, the proportions of glomerular glomerulosclerosis and S were critical predictors of poor prognosis of IgAN [14, 26, 27]. As expected, we confirmed that a low eGFR, heavy proteinuria and increased S were significantly associated with an increased risk in both male and female patients. Generally, no significant differences were observed in the associated risk factors or renal outcomes relative to gender in IgAN patients.

This study has several limitations. First, this was a single-centre retrospective study, with a median follow-up time of 48.6 (34.7, 62.7) months, therefore, we will continue to follow up with these patients. The current follow-up time was not long enough compared with the development of the disease. Second, treatment may play a role in the prognosis of IgAN, but the effects of therapeutic interventions could not be considered. Therapeutic regimes were flexible according to the physicians’ clinical decision making, and immunosuppression, in particular, was not standardized; thus, such unadjusted confounding impairs data interpretation. Therefore, further well-designed, multicentre, large cohort studies with longer, regular follow-ups are necessary to confirm these findings.

## Conclusions

In summary, our findings suggested that male IgAN patients presented with worse clinicopathological changes than female patients, but when we controlled for the confounding effect of renal function by eGFR- and Scr-matching using frequency matching analyses, no significant difference in long-term renal survival was observed between male and female patients.

## Abbreviations

ACEI: Angiotensin converting enzyme inhibitor
ARB: Angiotensin II receptor blocker
BP: Blood pressure
BUN: Blood urine nitrogen
CI: Confidence interval
CrCl: Creatinine clearance
E: Endocapillary hypercellularity
eGFR: Estimated glomerular filtration rate
EM: Electron microscopy
ESRD: End-stage renal disease
GFR: Glomerular filtration rate
HR: Hazard ratio
IgAN: Immunoglobulin A nephropathy
M: Mesangial hypercellularity
MAP: Mean arterial pressure
MDRD: Modification of diet in renal disease
S: Segmental sclerosis
Scr: Serum creatinine
SUA: Serum uric acid
T: Tubular atrophy/interstitial fibrosis

## References

[1] Wyatt RJ, Julian BA. IgA Nephropathy. N Engl J Med 2013; 368 (25): 2402–2414. 10.1056/NEJMra1206793 23782179

[2] Geddes CC, Rauta V, Gronhagen-Riska C, Bartosik LP, Jardine AG, Ibels LS, Pei Y, Cattran DC (2003). A tricontinental view of IgA nephropathy. Nephrol Dial Transplant.

[3] Donadio JV, Grande JP (2002). IgA Nephropathy. N Engl J Med.

[4] Duan ZY, Cai GY, Chen YZ, Liang S, Liu SW, Wu J, Qiu Q, Lin SP, Zhang XG, Chen XM (2013). Aging promotes progression of IgA nephropathy: a systematic review and meta-analysis. Am J Nephrol.

[5] Goto M, Wakai K, Kawamura T, Ando M, Endoh M, Tomino Y (2009). A scoring system to predict renal outcome in IgA nephropathy: a nationwide 10-year prospective cohort study. Nephrol Dial Transplant.

[6] Tan M, Li W, Zou G, Zhang C, Fang J (2015). Clinicopathological features and outcomes of IgA nephropathy with hematuria and/or minimal proteinuria. Kidney Blood Press Res.

[7] Natural D'AG (2004). History of idiopathic IgA nephropathy and factors predictive of disease outcome. Semin Nephrol.

[8] Riispere Ž, Laurinavičius A, Kuudeberg A, Seppet E, Sepp K, Ilmoja M, Luman M, Kõlvald K, Auerbach A, Ots-Rosenberg M (2016). IgA nephropathy clinicopathologic study following the Oxford classification: Progressionpeculiarities and gender-related differences. Medicina (Kaunas).

[9] Moriyama T, Tanaka K, Iwasaki C, Oshima Y, Ochi A, Kataoka H, Itabashi M, Takei T, Uchida K, Nitta K (2014). Prognosis in IgA nephropathy: 30-year analysis of 1,012 patients at a single center in Japan. PLoS One.

[10] Cattran DC, Reich HN, Beanlands HJ, Miller JA, Scholey JW, Troyanov S (2008). The impact of sex in primary glomerulonephritis. Nephrol Dial Transplant.

[11] Donadio JV, Bergstralh EJ, Grande JP, Rademcher DM (2002). Proteinuria patterns and their association with subsequent end-stage renal disease in IgA nephropathy. Nephrol Dial Transplant.

[12] Garovic VD, August (2016). *sex* Differences and renal protection: keeping in touch with your feminine side. J Am Soc Nephrol.

[13] Ma YC, Zuo L, Chen JH, Luo Q, Yu XQ, Li Y, Xu JS, Huang SM, Wang LN, Huang W, Wang M, Xu GB, Wang HY (2006). Modified glomerular filtration rate estimating equation for Chinese patients with chronic kidney disease. J Am Soc Nephrol.

[14] Cattran DC, Coppo R, Cook HT, Feehally J, Roberts IS, Troyanov S, Alpers CE, Amore A, Barratt J, Berthoux F (2009). The Oxford classification of IgA nephropathy: rationale, clinicopathological correlations, and classification. Kidney Int.

[15] Li PK, Ho KK, Szeto CC, Yu L, Lai FM (2002). Prognostic indicators of IgA nephropathy in the Chinese--clinical and pathological perspectives. Nephrol Dial Transplant.

[16] Le W, Liang S, Hu Y, Deng K, Bao H, Zeng C, Liu Z (2012). Long-term renal survival and related risk factors in patients with IgA nephropathy: results from a cohort of 1155 cases in a Chinese adult population. Nephrol Dial Transplant.

[17] Knoop T, Vikse BE, Svarstad E, Leh S, Reisæter AV, Bjørneklett R (2013). Mortality in patients with IgA nephropathy. Am J Kidney Dis.

[18] Eiro M, Katoh T, Sakuma Y, Sakurai K, Suzuki H, Asahi K, Watanabe K, Watanabe T (2003). Insulin Resistance highly associates with hypertension in IgA nephropathy. Clin Nephrol.

[19] Tanaka K, Moriyama T, Iwasaki C, Takei T, Nitta K (2015). Effect of hematuria on the outcome of IgA nephropathy with mild proteinuria. Clin Exp Nephrol.

[20] Pottel H, Hoste L, Delanaye P, Cavalier E, Martens F (2012). Demystifying ethnic/sex differences in kidney function: is the difference in (estimating) glomerular filtration rate or in serum creatinine concentration?. Clin Chim Acta.

[21] Wetzels JF, Kiemeney LA, Swinkels DW, Willems HL, den Heijer M (2007). Age- and gender-specific reference values of estimated GFR in Caucasians: the Nijmegen biomedical study. Kidney Int.

[22] Kurahashi H, Watanabe M, Sugimoto M, Ariyoshi Y, Mahmood S, Araki M, Ishii K, Nasu Y, Nagai A, Kumon H (2013). Testosterone replacement elevates the serum uric acid levels in patients with female to male gender identity disorder. Endocr J.

[23] Nagasawa Y, Yamamoto R, Shoji T, Shinzawa M, Hasuike Y, Nagatoya K, Yamauchi A, Hayashi T, Kuragano T, Moriyama T, Isaka Y, Nakanishi T (2016). Serum uric acid level predicts progression of IgA nephropathy in females but not in males. PLoS One.

[24] Takiue Y, Hosoyamada M, Kimura M, Saito H (2011). The effect of female hormones upon urate transport systems in the mouse kidney. Nucleosides Nucleotides Nucieic Acids.

[25] Kolz M, Johnson T, Sanna S, Teumer A, Vitart V, Perola M, Mangino M, Albrecht E, Wallace C, Farrall M (2009). Meta-analysis of 28,141 individuals identifies common variants within five new loci that influence uric acid concentrations. PLoS Genet.

[26] Alamartine E, Sauron C, Laurent B, Sury A, Seffert A, Mariat C (2011). The use of the Oxford classification of IgA nephropathy to predict renal survival. Clin J Am Soc Nephrol.

[27] Tanaka S, Ninomiya T, Katafuchi R, Masutani K, Tsuchimoto A, Noguchi H, Hirakata H, Tsuruya K, Kitazono T (2013). Development and validation of a prediction rule using the Oxford classification in IgA nephropathy. Clin J Am Soc Nephrol.

